# Racial Differences in Chronic Pain and Quality of Life among Adolescents and Young Adults with Moderate or Severe Hemophilia

**DOI:** 10.1007/s40615-015-0107-x

**Published:** 2015-04-03

**Authors:** John M. McLaughlin, Angela Lambing, Michelle L. Witkop, Terry L. Anderson, James Munn, Bartholomew Tortella

**Affiliations:** US Medical Affairs, Pfizer Inc, New York, NY USA; Henry Ford Adult Hemophilia & Thrombosis Treatment Center, Detroit, MI USA; Northern Regional Bleeding Disorders Center, Traverse City, MI USA; University of Michigan Hemophilia Treatment Center, Ann Arbor, MI USA

**Keywords:** Clotting factor, Hemophilia, Quality of life, Pain, Adherence, Racial disparity, Race

## Abstract

**Background and Objective:**

We explored racial differences in adherence to recommended clotting factor treatment regimens, chronic pain, and quality of life (QoL) among adolescents and young adults (AYAs) diagnosed with moderate or severe hemophilia.

**Methods:**

A convenience sample of hemophilia patients aged 13–25 years completed an online cross-sectional survey in 2012. Chronic pain was measured using the revised Faces Pain Scale (FPS-R) and dichotomized as *high* (FPS-R ≥ 4) or *low* (FPS-R < 4). QoL was measured with the SF-36.

**Results:**

Of 80 AYA participants (79 male), most had severe disease (91 %) and hemophilia A (91 %). Most were white (76 %) and non-Hispanic (88 %). At the univariate level, compared to whites, non-whites were more likely to have produced an inhibitor against clotting factor treatment (74 vs 38 %, *p* < .01), less likely to have commercial health insurance (16 vs 63 %, *p* < .001), more likely to report high levels of chronic pain (FPS-R ≥ 4) (63 vs 26 %, *p* < .01), and had lower SF-36 physical composite summary (PCS) scores. Adjusted logistic and quantile regression modeling, respectively, revealed that non-whites were 5.31 (95 % CI 1.62, 17.4; *p* < .01) times more likely to report high chronic pain and had median PCS scores that were 26.0 (95 % CI 11.0, 40.9; *p* < .01) points lower than whites.

**Conclusions:**

Targeted efforts to prevent and manage chronic pain among non-white AYAs with moderate or severe hemophilia are necessary. After accounting for demographic and clinical differences, there were no racial differences in adherence to recommended clotting factor treatment regimens; however, non-whites were more than five times more likely to report high levels of chronic pain, which predicted worse overall physical QoL, bodily pain, physical and social functioning, and greater role limitations due to physical health.

## Background

Chronic pain is a known complication of persistent hemophilia joint hemarthrosis. Despite being both a pervasive problem among persons with hemophilia (PWH) and a well-known predictor of general quality of life (QoL) [1–5], there are remarkably few studies that have evaluated the prevalence and management of chronic pain and the impact of chronic pain on QoL among PWH. What few studies do exist have demonstrated that chronic pain is suboptimally prevented [6] and treated in PWH [2, 4, 7]. Specifically, previous research has shown that more than one third of adults and nearly 1 in 10 children with hemophilia reported having chronic pain [2] and that 39 % of adult PWH reported their pain was not well managed [4].

Although this limited amount of research has provided some insight into chronic pain among PWH, no studies have evaluated whether racial differences exist in the development and management of chronic pain among PWH. Studies describing racial differences in chronic pain related to hemophilia are warranted given that previous research in other disease areas has shown that racial minorities, particularly African-Americans, report less pain tolerance/coping [8, 9], higher overall levels of pain [8, 10–13], less control over pain [8, 10], and more sequelae of chronic pain including worse QoL [9], higher rates of depression [10, 12], more disability [13, 14], and, in severe cases, the development of post-traumatic stress disorder and other chronic pain syndromes [13]. These racial differences have been shown to persist across all types of pain [15] and across a variety of settings, including acute pain managed in the emergency department, postoperative care, childbirth, chronic non-cancer pain, arthritis pain, cancer-related pain, and end-of-life care [16].

Adolescent and young adult (AYA) PWH are a subpopulation for which data describing chronic pain, QoL, and racial disparities are particularly lacking. AYAs are a unique population of PWH who are often not only just starting to take more responsibility for the management of their own disease but also developing treatment habits that can carry over into adult life [17]. Thus, developing proper pain management strategies early on with AYA PWH is especially important. Previous research, though not in PWH, has shown that younger individuals report more depressive symptoms and greater pain intensity and are less successful at coping with pain [18]. Therefore, building strong pain management habits for AYA PWH may be especially important in times during and just before young adulthood, since it may be a time when coping with pain is the most difficult. Our previous study discovered that racial disparities in chronic pain may exist among AYA with moderate or severe hemophilia and that a further, more detailed analysis was warranted [6]. To that end, the primary aim of this study was to evaluate racial/ethnic differences in adherence to recommended clotting factor treatment regimens, chronic pain, and QoL among AYAs with moderate or severe hemophilia using data collected via a large cross-sectional survey administered in 2012.

## Methods

### Study Population and Recruitment

Data describing self-reported adherence to recommended treatment regimens, level of chronic pain, and QoL among AYA persons with a bleeding disorder were obtained as part of the larger Interrelationship between Management of Pain, Adherence to Clotting factor Treatment, and Quality of Life (IMPACT QoL) survey. As the name suggests, the IMPACT QoL study had the primary goal of assessing the relationship between validated measures of pain, clotting factor adherence, and QoL among AYA PWH and AYAs with von Willebrand disease (VWD). Data were collected via a one-time, cross-sectional, online survey from a convenience sample of AYA persons with a bleeding disorder. To be eligible to complete the survey, participants had to i) be aged 13–25 years, ii) read, write, and speak English, and iii) have hemophilia A, hemophilia B, or VWD. Recruitment occurred at major US hemophilia meetings (e.g., Inhibitor Summits and National Hemophilia Foundation meetings), US hemophilia treatment centers (HTC), and through a Facebook™ (Facebook, Menlo Park, CA, USA) page dedicated to the study from April through December of 2012. All surveys were completed electronically using SurveyMonkey™ (SurveyMonkey, Palo Alto, CA, USA) and Apple iPads™ (Apple, Cupertino, CA, USA). The study was approved by the Munson Medical Center (Traverse City, MI) institutional review board prior to data collection. All data were de-identified prior to analysis.

The current study uses a patient subset (AYAs with moderate or severe hemophilia) of the IMPACT QoL survey data to analyze the relationship between race and three major factors related to the adequate management of hemophilia: i) adherence to prescribed clotting factor treatment regimen, ii) chronic pain, and iii) QoL. Participants with mild hemophilia or VWD were excluded from this analysis because of small sample size and to minimize heterogeneity in the assessment of the primary hypothesis (i.e., those with moderate and severe hemophilia are at greatest risk of joint disease and chronic pain resulting from hemarthroses).

### Measurement

Race was self-reported as white, black or African-American, Asian, American Indian or Alaskan Native, or mixed race. Due to small sample size for non-white racial categories and for purpose of analysis, race was dichotomized as white (only) vs non-white. In addition to racial classification, we also collected information about ethnicity (Hispanic vs non-Hispanic). For purposes of analysis, racial and ethnic categories were treated as two separate dichotomous variables; however, we did conduct sensitivity analyses to determine if results were similar for a combined racial/ethnic category (non-Hispanic white vs non-white or Hispanic).

Adherence to recommended clotting factor treatment regimens was assessed using the Validated Hemophilia Regimen Treatment Adherence Scale (VERITAS)-Pro [19] for participants who reported treating their bleeding disorder prophylactically. The VERITAS-PRN [20] was used to measure adherence for participants who reported on-demand (i.e., episodic) factor treatment. VERITAS scores range from 24 (most adherent) to 120 (least adherent). As an experimental measure, we also combined VERITAS-Pro and VERITAS-PRN responses into one category (VERITAS-combined) [21] to evaluate the relationship between adherence and QoL for both prophylactic and on-demand AYA PWH simultaneously. The cutoff for non-adherent prophylactic participants was a total VERITAS-Pro score ≥57 as previously established [19]. The cutoff for non-adherent on-demand patients has not been previously described and was defined as those with VERITAS-PRN in the highest quartile of all responses. This value was chosen because the VERITAS-Pro cutoff was approximately the 75th percentile of all responses.

Chronic pain was measured using the revised Faces Pain Scale (FPS-R). The FPS-R is a visual scale composed of six faces illustrating an increasing level of pain intensity. Respondents were asked to choose the face that best describes the intensity of the chronic pain they experienced. In the IMPACT QoL survey, chronic pain was defined as “pain that you have every day or almost every day and that always or almost always seems to be there even when you are not having a bleed at that moment.” FPS-R scores range from zero to 10 (in increments of two) with the faces representing the lowest and highest levels of pain intensity coded as zero and 10, respectively. The FPS-R is highly correlated with the visual analog scale (*r* = .93) and with the colored analog scale (*r* = .84), demonstrating strong validity. Reliability and validity of the FPS-R have been established for a broad age range, ranging from children as young as 4 years old to adults [22]. For purpose of analysis, chronic pain was dichotomized as *high* for those who reported their pain as “4—moderate,” “6—severe,” “8—very severe,” or “10—worst pain possible” (i.e., FPS-R ≥ 4) and *low* for “2—mild pain,” or “0—no pain” (i.e., FPS-R < 4). In addition, other, non-validated pain-related measures were collected to assess feelings of control over pain, use of prescribed pain medication, and how well health care providers both managed and listened to participants about their pain.

QoL was measured using the 36-Item Short Form Health Survey (SF-36). The SF-36 is composed of 8 multi-item scales (35 items) assessing i) bodily pain (BP, 2 items), ii) physical function (PF, 10 items), iii) role limitations due to physical health problems (RP, 4 items), iv) general health (GH, 5 items), v) vitality (VT, 4 items), vi) social functioning (SF, 2 items), vii) role limitations due to emotional problems (RE, 3 items), and viii) emotional well-being/mental health (MH, 5 items) [23]. The 36th item, which asks about health change, is not included in the scale or summary scores. Each SF-36 subscale is calculated by standardizing the average of the participant’s responses to the questions contained in the subscale, so that each subscale has a final range of 0 (lowest level of functioning) to 100 (highest level of functioning). These eight scales were then aggregated into two summary measures: the Physical (PCS) and Mental (MCS) Component Summary scores, with higher scores representing better health. The scoring algorithm for PCS includes positive weights for the PF, RP, BP, GH, and VT scales and negative weights for the SF, RE, and MH scales. The scoring algorithm for MCS includes positive weights for the VT, SF, RE, and MH scales and negative weights for the PF, RP, BP, and GH scales. PF, RP, and BP contribute most to the scoring of the PCS. MH, RE, and SF scales contribute most to the scoring of the MCS. Three of the scales (VT, GH, and SF) have noteworthy correlations with both components. The SF-36 was constructed for self-administration by persons 14 years of age and older and for administration by a trained interviewer in person or by telephone [23].

Other self-reported data collected included information about participant age, gender, ethnicity, health insurance status/type, and the educational level of the participants’ parents. Data were also collected about bleeding disorder type (hemophilia A or B), whether or not the participant ever produced an inhibitor against clotting factor treatment, and bleeding disorder severity. For hemophilia A and B, severity was classified as mild, moderate, or severe corresponding to 6–50 %, 1–5 %, and <1 % factor VIII activity, respectively.

### Statistical Analysis

Descriptive statistics and univariate relationships were assessed by tabulating race (white vs non-white) by participant sociodemographic and clinical characteristics and by outcomes of interest. Percentages were used to describe categorical variables and statistical association was assessed using Fisher’s exact test because of small sample size. Because of the skewed nature of adherence, chronic pain, and QoL measures, medians were used to describe these outcomes when assessed as continuous variables. Statistically significant differences across continuous variables were assessed using the nonparametric Wilcoxon rank sum test or Kruskal-Wallis test (depending on degrees of freedom), as they do not assume a normal distribution of the residuals.

Multivariable logistic regression analysis was used to predict clotting factor treatment non-adherence (vs adherence) based on the VERITAS cutoff described previously. To assess chronic pain, multivariable, logistic regression models were constructed to assess factors associated with having high (vs low) levels of chronic pain. Because QoL data were largely skewed, multivariable, quantile regression models were used to assess factors associated with SF-36 PCS and MCS subscales. For both logistic and quantile regression models due to the large number of variables collected as part of the survey and because of the small sample size inherent in rare disease research, in addition to the fully adjusted models, final parsimonious models were constructed. In the final parsimonious models, we decided, a priori, to model race (non-white vs white) as the primary variable of interest and include other covariates in the model only if they i) changed the odds ratio (OR) of the race parameter by at least 10–15 % (i.e., confounded) [24], ii) improved the precision of the estimated race parameter, or iii) were statistically significant at a two-tailed alpha level of .05. All statistical analyses were performed using SAS 9.2 (SAS Institute Inc., Cary, North Carolina) and STATA 12 (StataCorp LP, College Station, Texas). All *p* values were calculated using two-sided tests. As this was an exploratory analysis, no adjustments for multiple comparisons were made.

## Results

Eighty AYAs with moderate or severe hemophilia completed the IMPACT QoL survey. The participants were predominantly male (*n* = 79), nearly all had severe disease (91 %) and hemophilia A (91 %). Roughly half were between the ages of 13–17 years (51 %), and most were never married (93 %) and had some type of health insurance (94 %). Of the 80 participating AYAs, 61 (76 %) self-reported their race as white, 14 (18 %) were black or African-American, 2 (3 %) were Asian, 2 (3 %) were mixed race, and 1 participant was American Indian or Alaskan Native. Overall, 13 % (10/80) of participants were Hispanic.

At the univariate level, compared to whites, non-whites were more likely to have produced an inhibitor against clotting factor treatment (74 vs 38 %, *p* < .01) and less likely to have commercial health insurance (16 vs 63 %, *p* < .001) (Table 1). Race had little impact on adherence (Table 2); however, compared to whites, non-whites were more likely to report high levels of chronic pain (FPS-R ≥ 4) (63 vs 26 %, *p* < .01) and had higher median chronic pain FPS-R scores (4 vs 0, *p* = .01) (Table 2). Acute pain FPS-R scores at the time of having a bleed were also higher for non-white participants (6 vs 4, *p* = .07), though it did not reach statistical significance. Other, non-validated pain-related measures suggested that there were no racial differences in feeling in control of pain, use of prescribed pain medication, or in how well health care providers both managed and listened to participants about their pain (Table 3). Compared to whites, QoL scores were statistically significantly lower for non-whites for PCS, BP, PF, RP, and SF components of the SF-36 (Table 2).

**Table 1:** Self-reported respondent characteristics by race among adolescents and young adults with moderate or severe hemophilia (*n* = 80)

Participant characteristic	Non-white^a^, *n* (%) (*n* = 19)		White, *n* (%) (*n* = 61)		Fisher’s *p* value
Age					.80
13–17	9	(47)	32	(52)	
18–25	10	(53)	29	(48)	
Gender					.24
Male	18	(95)	61	(100)	
Female	1	(5)	0	(0)	
Ethnicity					1.0
Hispanic	2	(14)	8	(12)	
Non-Hispanic	17	(86)	53	(88)	
Health Insurance^b^					<.001
Medicaid or VA only^c^	12	(63)	12	(20)	
Commercial only	3	(16)	32	(52)	
Both	0	(0)	7	(11)	
Insured—type unknown	1	(5)	8	(13)	
Uninsured	3	(16)	0	(0)	
Mother’s education					.78
Less than bachelor’s	14	(74)	42	(69)	
Bachelor’s or higher	5	(26)	19	(31)	
Father’s education					.16
Less than bachelor’s	16	(84)	39	(64)	
Bachelor’s or higher	3	(16)	22	(36)	
Bleeding disorder					.19
Hemophilia A	19	(100)	54	(89)	
Hemophilia B	0	(0)	7	(11)	
Severity					1.0
Moderate	1	(5)	6	(10)	
Severe	18	(95)	55	(90)	
Inhibitor development					<.01
Never	5	(26)	38	(62)	
Ever	14	(74)	23	(38)	
Treatment regimen					.72
On-demand	3	(16)	8	(13)	
Prophylaxis	16	(84)	53	(87)	

^a^Most (73 %) non-white respondents were black or African-American, 14 % were mixed race, 9 % were Asian, and 5 % were American Indian or Alaskan Native

^b^ *n* = 78, two patients answered “Don’t Know” to whether or not they had health insurance and were not included

^c^Only two patients had VA only insurance; the others had Medicaid only

**Table 2:** Self-reported outcomes by race among adolescents and young adults with moderate or severe hemophilia (*n* = 80)

	Non-white^a^(*n* = 19)		White (*n* = 61)		
Outcome of interest	Median	Mean	Median	Mean	*p* value^d^
Adherence^b^					
VERITAS-Pro	47.5	48.4	48.0	50.0	.87
VERITAS-PRN	43.0	47.3	52.0	52.4	.41
VERITAS-combined	47.0	48.3	49.0	50.3	.69
Chronic pain^c^					
FPS-R	4.0	3.9	0	1.8	.01
Acute pain^c^					
FPS-R	6.0	5.9	4.0	4.7	.07
QoL (SF-36)					
PCS	59.0	63.4	83.8	78.0	.02
MCS	75.0	71.3	76.4	73.3	.62
BP	62.5	60.5	80.0	74.8	.02
PF	55.0	63.7	90.0	79.8	.03
RP	75.0	61.8	100.0	79.5	.01
GH	70.0	65.3	75.0	74.6	.21
VT	65.0	64.2	60.0	61.1	.72
SF	75.0	71.7	87.5	82.2	.05
RE	100.0	73.7	100.0	80.3	.55
MH	76.0	75.4	76.0	75.3	.96

*PCS* the physical composite summary score, *MCS* the mental composite summary score, *BP* bodily pain (2 items), *PF* physical function (10 items), *RP* role limitations due to physical health problems (4 items), *GH* general health (5 items), *VT* vitality (4 items), *SF* social functioning (2 items), *RE* role limitations due to emotional problems (3 items), *MH* emotional well-being/mental health (5 items)

^a^Most (73 %) non-white respondents were black or African-American, 14 % were mixed race, 9 % were Asian, and 5 % were American Indian or Alaskan Native

^b^Adherence was assessed using the Validated Hemophilia Regimen Treatment Adherence Scale (VERITAS)-Pro for participants who reported treating their bleeding disorder prophylactically (*n* = 69). The VERITAS-PRN was used to measure adherence for participants who reported on-demand (i.e., episodic) factor treatment (*n* = 11). Possible VERITAS subscale scores ranged from 4 points (most adherent) to 20 (least adherent), and possible total scores ranged from 24 (most adherent) to 120 (least adherent). As an experimental measure, we also combined VERITAS-Pro and VERITAS-PRN responses into one category (VERITAS-combined) to evaluate the relationship between adherence and QoL for both prophylactic and on-demand AYA PWH simultaneously

^c^FPS-R is the revised Faces Pain Scale. FPS-R scores range from zero to 10 (in increments of two) with the faces representing the lowest and highest levels of pain intensity coded as zero and 10, respectively

^d^ *p*-values were calculated by comparing the difference in medians by age group using the Wilcoxon rank sum test

**Table 3:** Self-reported, non-validated secondary pain outcomes by race among adolescents and young adults with moderate or severe hemophilia (*n* = 80)

Non-validated pain measures	Non-white^a^ (*n* = 19)		White (*n* = 61)		Fisher’s *p* value^e^
	*n*	(%)	*n*	(%)	
Do you feel in control of your pain?					
I control my pain	10	(53)	42	(69)	.35
I cannot decide	7	(37)	12	(20)	
My pain controls me	2	(11)	7	(11)	
Do you take more pain medication (higher dosage or more pills) than prescribed?					
Yes	7	(37)	11	(18)	.09
No	12	(63)	50	(82)	
Do you take pain medication more often (prescribed dosage but at more frequent intervals) than prescribed?					
Yes	5	(26)	15	(25)	1.0
No	14	(74)	46	(75)	
How often do you take your pain medication?					
Every day, almost every day, or on a regular, scheduled basis	8	(42)	15	(25)	.16
Only when needed	11	(58)	46	(75)	
Excluding clotting factor, do you take additional pain medication when you are having a bleed?					
Always, often, or sometimes	11	(58)	40	(66)	.59
Never or rarely	8	(42)	21	(34)	
How well do you think your doctor/provider treats/manages your pain?					
Excellent or very well	13	(68)	41	(67)	1.0
Well, fair, or poor	6	(32)	20	(33)	
How well do you think your doctor/provider listens to you about your pain?					
Excellent or very well	15	(79)	42	(69)	.56
Well, fair, or poor	4	(21)	19	(31)	

^a^Most (73 %) non-white respondents were black or African-American, 14 % were mixed race, 9 % were Asian, and 5 % were American Indian or Alaskan Native

Adjusted logistic regression modeling revealed that non-white AYAs were 5.31 (95 % CI 1.62, 17.4, *p* < .01) times more likely to report high chronic pain compared to whites (Table 4). Adjusted quantile regression modeling showed that non-whites had median PCS scores that were 26.0 (95 % CI 11.0, 40.9, *p* < .01) points lower than whites when chronic pain was excluded from the model. However, after statistical adjustment for chronic pain level, the difference in PCS between whites and non-whites disappeared (Table 5), suggesting that racial differences in chronic pain level accounted for racial differences in physical QoL. Sensitivity analyses showed that results were similar if race and ethnicity were treated as a combined racial/ethnic category (non-Hispanic white vs non-white or Hispanic), rather than two separate variables.

**Table 4:** Logistic regression analyses evaluating the relationship between race (Non-white vs white) and self-reported high (vs low) levels of chronic pain among adolescents and young adults with moderate or severe hemophilia (*n* = 80)

Model	OR	95 % CI	*p* value
Crude	4.82	1.62, 14.4	<.01
Fully adjusted^a^	4.85	1.32, 17.8	.02
Parsimonious^b^	5.31	1.62, 17.4	<.01

^a^In addition to non-white vs white, the fully adjusted model includes age, ethnicity, health insurance status, mother’s education level, father’s education level, history of inhibitor development, bleeding disorder type, bleeding disorder severity, VERITAS-combined score, and history of inhibitor development

^b^In addition to non-white vs white, the final parsimonious model includes VERITAS-combined score and history of inhibitor development

**Table 5:** Quantile regression analyses evaluating the relationship between race (Non-white vs white) and SF-36 physical composite score (PCS) among adolescents and young adults with moderate or severe hemophilia (*n* = 80)

Model	Coef.	95 % CI	*p* value
Crude	−24.8	−40.6, −8.92	<.01
Fully adjusted without chronic pain^a^	−26.0	−40.8, −11.1	<.01
Fully adjusted with chronic pain^a^	0.71	−11.0, 9.56	.89
Parsimonious without chronic pain^b^	−26.0	−40.9, −11.0	<.01
Parsimonious with chronic pain^b^	2.38	−13.9, 9.2	.68

^a^In addition to non-white vs white, the fully adjusted model includes age, ethnicity, health insurance status, mother’s education level, father’s education level, history of inhibitor development, bleeding disorder type, bleeding disorder severity, VERITAS-combined score, and history of inhibitor development

^b^In addition to non-white vs white, the final parsimonious model includes age, ethnicity, and history of inhibitor development

## Discussion

Results from our study showed that, compared to white AYA with moderate or severe hemophilia, non-whites were more than five times as likely to experience moderate-to-severe chronic pain (FPS-R ≥ 4) and that worse chronic pain, in turn, was related to worse overall physical QoL, bodily pain, physical and social functioning, and greater role limitations due to physical health. These findings are particularly concerning because they persisted even after statistical adjustment for demographic (e.g., age, ethnicity, health insurance status, and parent’s education level) and clinical factors (e.g., bleeding disorder severity and type, history of inhibitor development, and type of and adherence to recommended clotting factor treatment regimen). Previous research, though not specific to PWH, supports our findings and has shown that African-American children have significantly higher postoperative pain scores and require more morphine than white children [25]. A systematic review of racial disparities in pain management suggested that significant racial and ethnic disparities exist in both evaluating pain and providing adequate analgesics. Specifically, racial/ethnic minorities were more likely to experience greater pain and worse pain management across a variety of health care settings including acute pain managed in the emergency department, postoperative care, childbirth, chronic non-cancer pain, arthritis pain, cancer-related pain, and end-of-life care [16]. Additionally, previous research has suggested that compared to whites, black or African-American patients with chronic pain experience greater pain severity, more disability, and heightened psychosocial problems related to their pain, including depression, post-traumatic stress disorder, and anxiety [13, 26]. We have been able to confirm many of the findings from these previous studies in a population (AYA PWH) for whom, to our knowledge, no data exist describing racial differences in pain and overall QoL.

Racial differences in pain and QoL discovered in our study, and in previous research, could partially be driven by biologic and genetic differences. It has long been identified that genetic factors may play a role in the perception and response to pain [27]. It has been previously suggested that genetic differences could influence activity in the higher nervous centers that modulate pain through the activation of the descending and neural inhibitory controls of African-Americans [28], which could contribute to enhanced pain sensitivity to noxious stimuli [8]. Others have postulated that differences in pain could be due in part to potential differences in circulating B-endorphins in response to stress among differing racial groups [29]. Moreover, significant variations in the allele frequency for multiple gene loci among racial groups have been identified that modulate nociceptive transduction, opioid analgesic effects, and neurotransmitter metabolism [16]. Other research has suggested that endogenous pain regulatory mechanisms in African-Americans may contribute to greater rates of clinical pain symptoms [30, 31]. Finally, consistent with previous research [32–38], our results showed that non-white AYA PWH were much more likely to have ever produced an inhibitor against clotting factor treatment. Though reasons for this are unknown, one possibility may be that a greater degree of population-level variation exists in factor VIII amino acid sequence among non-white persons, leading to an increased probability of a mismatch with replacement factor proteins [39, 40]. Other studies, however, have suggested that variations in factor VIII genes do not explain higher inhibitor frequencies in African-American and Hispanic patients [41] and that genetic factors other than the disease-causing mutation could play a role [34, 42, 43]. Another report suggested that patients receiving plasma-derived activated prothrombin complex concentrates were more likely to be African-American [44], which may suggest that our inhibitor (ever vs never) designation may not fully control for the impact of inhibitor development.

Secondly, social and cultural factors may drive racial differences. It has been previously suggested that differences in provider cultural beliefs and stereotypes regarding pain in minority individuals affects the prescribing of opioid analgesia [45–48] and may even indirectly influence provider decision-making and the overall content and tone of the clinical encounter [49–52]. It is important to note, however, that we did not find any significant differences (all *p* > .55) across racial groups in how well participants thought their health care provider treated and managed their pain or in how their health care provider listened to them about their pain—though validated measures were not used to address those specific aspects of provider pain management in our survey. In addition to potential conscious and unconscious health care provider bias against racial minorities, there is also the possibility that racial minorities have heightened sensitivity to being labeled as complainers or are afraid that pain means a progression of illness, and thus may underreport pain to their health care providers [53].

Language and/or health literacy barriers may exist among certain racial/ethnic groups, limiting appropriate assessment and treatment of pain by medical providers. This may lead to an underestimation of pain severity in minority patients, which, in turn, may result in minority populations being less likely to receive appropriate pain management [45]. Furthermore, the degree of cultural affiliation and acculturation may also have a relationship to the pain response among varying racial/ethnic groups [8]. Pain responses may also differ based upon the culturally appropriate strategies utilized for reducing pain. Designing culturally appropriate treatment strategies should include personally delivered interventions that take into account cultural practices, philosophies, products, and/or appropriate environment vehicles to facilitate behavior change [16].

A third potential explanation for racial differences in chronic pain revolves around psychological issues—primarily beliefs and coping strategies. Previous research suggests that African-Americans use emotion-focused coping strategies more than whites [53]. Minority patients have been reported to display stoicism, describe pain as “inevitable,” have a fear of addiction to pain medications, and express concerns of intolerable side effects [54]. Generally speaking, minority patients rely more on alternative and complementary pain treatments [16] and are more likely to use prayer and other religious coping strategies [15, 16, 18, 28], perhaps due to a mistrust in the medical community and inadequate medical treatment for pain relief [28]. Indeed, there may be systematic differences in psychological distress and pain-coping strategies across racial groups [55].

Final factors that may affect the disparity in pain treatment among minorities include health care system-related variables. It is well known that racial/ethnic minorities and the poor have decreased access to health care [16] which translates to more limited access to pain treatment [56]. Even if minority patients receive a prescription for pain medication, they may face additional structural barriers such as limited availability of opioids in their neighborhood pharmacy, lack of adequate health insurance to pay for their prescription, or financial difficulties in paying for health care despite having health insurance [57]. In this study, however, race/ethnicity had little impact on adherence to prescribed clotting factor treatment regimen.

This study has limitations. Primarily, data were cross-sectional; thus, causal inference cannot be made. Temporality, however, is not problematic when assessing the effect of race/ethnicity on health outcomes. A second limitation is that all data are self-reported. As such, information about blood disorder type and severity, health insurance coverage, and other demographic, clinical, and behavioral information are not confirmed by medical record review or administrative claims data. However, by obtaining data through self-report, this study was able to collect important, reliable, and valid patient-reported outcome data about adherence and chronic pain. Patient-reported data about racial categories and pain are considered the “gold standard,” and the VERITAS scales are the only validated measures of adherence to clotting factor treatment developed to date—though we did combine them into one experimental measure for modeling purposes. Another limitation is that we were only able to dichotomize race as white vs non-white, as we did not have sufficient sample size to evaluate differences in racial disparities depending on specific, individual racial groups. Sensitivity analyses, however, suggested that ethnic disparities may exist in addition to racial disparities, as results were similar whether or not Hispanic whites were included in the “white only” category or not. Future studies should try to examine differences across individual racial/ethnic groups with sufficient statistical power. In addition, because of the way we evaluated inhibitor development (ever vs never), there may be residual confounding with respect to racial differences in inhibitor development, inhibitor type (transient vs persistent), bleeding patterns, and/or target joint development which was not captured in our study and should be examined in future research. Finally, AYA PWH were primarily recruited from large national or regional hemophilia meetings. Thus, our convenience sample of AYA PWH may not adequately represent the broader AYA PWH population who do not typically attend these meetings.

## Conclusion

Results showed that non-white AYA patients with moderate or severe hemophilia were more than five times as likely as whites to report high levels of chronic pain and that worse chronic pain, in turn, led to non-whites having worse overall physical QoL, bodily pain, physical and social functioning, and greater role limitations due to physical health. These findings are particularly concerning because they persisted even after statistical adjustment for demographic and clinical factors. Intrinsically, self-reported racial classifications are not particularly meaningful and are largely arbitrarily and socially constructed. Future research should more thoroughly examine the complex interplay between biological and genetic, social and cultural (including bias and discrimination), psychological and emotional, and health care system-related factors that could all contribute to these racial/ethnic disparities in chronic pain management and QoL. These factors, either alone or in combination, are the building blocks for the construction of racial/ethnic disparities and also represent the foundation for which programs addressing disparate health outcomes for racial/ethnic groups should be built. AYAs will remain an especially important target for this research, given that previous studies have shown that, regardless of race or ethnicity, compared to older adults, AYAs are less likely to have high pain thresholds, develop effective pain-coping skills, and adequately manage their pain [18].
